# The Illness Perceptions and Coping Experiences of Patients with Colorectal Cancer and Their Spousal Caregivers: A Qualitative Study

**DOI:** 10.3390/healthcare12111073

**Published:** 2024-05-24

**Authors:** Yi Zhang, Ye Wang, Rongyu Li, Zheng Sun, Qiuping Li

**Affiliations:** Wuxi School of Medicine, Jiangnan University, Wuxi 214122, China; 6222807091@stu.jiangnan.edu.cn (Y.Z.); 6222807063@stu.jiangnan.edu.cn (Y.W.); 6222807030@stu.jiangnan.edu.cn (R.L.); 6222807050@stu.jiangnan.edu.cn (Z.S.)

**Keywords:** colorectal cancer, illness perception, coping, qualitative study, spousal caregivers

## Abstract

(1) Background: Illness perception (IP) is an important psychological construct for couples dealing with cancer, which impacts health outcomes and the psychological adjustment to cancer. More research is needed to explore the traits of IP and the efforts of couples coping with cancer. Thus, this study was designed to explore the coping experiences and features of the IPs of couples dealing with cancer. (2) Methods: A total of 24 patients with colorectal cancer (CRC) and 20 spousal caregivers (SCs) participated in semi-structured interviews. All interviews were recorded digitally, transcribed, and analyzed by using an inductive thematic analysis. (3) Results: Two themes (individualized and predominant IP; IP sharing and restructuring) were developed. A preliminary framework was formulated to illustrate the relations among subthemes and the relations between themes with an adjustment of a positive IP to CRC. In this framework, based on multiple sources and factors, the natural disparities formed the IPs of the partners of couples and determined the incongruence of IPs. The effects of IP incongruence on lives under the disease guided the three directions of coping approaches (i.e., information and available support, appropriate disclosure and reflection, and leaving the CRC diagnosis behind) which were adopted by couples dealing with CRC to share and restructure the IP with their spouses for effective dyadic coping. (4) Conclusions: This study provides insights to healthcare providers into the experiences of couples dealing with CRC and the development of couple-based IP intervention programs: (a) it initially provides adequate factual knowledge for enhancing beliefs in the ability to control illness, (b) encourages illness-centered conversations and disclosure regarding thoughts and emotions for promoting positive congruence of IP between the partners of couples dealing with a hard dilemma, and (c) guides couples to perceive positive changes and explore the illness’s meaning. Understanding each theme of personalized IP and adopting effective IP coping approaches can help guide couples dealing with CRC to efficiently promote constructive IP and better health outcomes.

## 1. Introduction

According to statistical data from 2022, colorectal cancer (CRC) is the world’s third most common cancer and is ranked as the second most common cancer in China [1]. Except for the increasing incidence and morbidity, it is worth noting that the 5-year survival rate of patients with CRC has extended year by year, rising to 65% [2], which creates a tremendous cancer burden. 

The diagnosis and treatment of CRC often bring severe psychological and physical issues to patients, such as fear, stigma, depression, pain, and sexual dysfunction [3,4,5]. CRC also influences the primary caregivers of patients, especially spousal caregivers (SCs), imposing psychological distress, role conflicts, and caregiver burden [6,7]. While confronting CRC’s threat, SCs and patients jointly react to the cancer-related challenge and multifaceted variations in long-term survivorship, forming an “emotional system” [8]. The focus of the majority of studies has shifted from the individual level to the dyadic level of cancer patient (CP)–SC dyads [9,10,11]. Couple-based interventions showed positive effects in facilitating the quality of life (QOL), anxiety, depression, dyadic coping levels, post-traumatic growth, and self-efficacy [12,13,14].

While jointly coping with the disease, patients with CRC and SCs have to take measures based on their understanding and feelings to change their coping behaviors and adjust to the disease treatment and long-term influence, where the way a person perceives the disease motivates this process and impacts the treatment outcomes. Hence, it is necessary to understand the way they perceive CRC and their beliefs/feelings rather than directly providing support and neglecting their individualized psychological patterns.

Illness perception (IP) is an individual’s cognitive appraisal of illness. IP reflects individual understandings and explanations about a current disease or current symptoms, including cognitive and emotional perceptions, based on preserved knowledge and disease experiences. The vital significance and influence of IP in psychological research in the rehabilitation of patients with malignancy has also been gradually recognized and explored in recent years [15,16,17,18], including CRC [19,20]. 

The IPs of patients have verified prominent correlations with active coping [19], health behavior [18,21], and the QOL [22,23], as well as their caregivers [24]. For instance, a positive IP alleviated the perceived pressure and psychological burden to an extent, where the CPs more easily to constructed good expectations towards the subsequent treatment efficacy and became actively positive in coping with difficulty [19]. The findings of a prospective observational study verified that helping patients control the perceived symptoms and enhancing their beliefs about the treatment that could control their disease have become potential methods to prevent long-lasting depression [25]. While the patients perceived the disease and treatment pessimistically and were in the grip of negative emotions, they felt higher levels of cancer-related fatigue and could not be conscious of the benefits of experiencing the disease, and they had low-level QOL [23]. This may be due to the fact that a negative IP generated the belief that symptoms were untreatable and may have prevented patients from seeking health care support [26].

SCs obtained inadequate care support and enormous information that may have gone beyond their ability to cope [27]. The research findings even revealed that SCs experienced higher levels of maladaptive IPs compared with the patients they were caring for [27]. In particular, evidence [27] has uncovered a mismatch of IPs between patients with CRC and SCs, which impacted their QOL. CPs tended to weaken the concept of cancer and move forward, whereas SCs perceived the long timeline of cancer and treated cancer as a permanent life-changing event [27]. The dissimilarity and mutuality of IP between CPs and SCs could negatively or positively impact dyadic coping, anxiety, depression, and QOL [11,24].

Evidence shows that IP acts as a valuable psychological variable while attempting to improve the well-being of both CPs [23] and caregivers of patients with cancer [28]. Thus, to further improve the health and well-being of patients with CRC and SCs, health promoters should emphasize and utilize the mediating effects of IPs when providing psychological support [29].

However, through an extensive review, it was found that there is no research focused on improving the IP of CPs and SCs. Few studies were constructed to improve the IPs, QOL, psychological outcomes, and physical outcomes of patients with diverse cancer types, which mainly provided emotional and information support through face-to-face or web-based interventions, but there lacks a tailored intervention program for CRC populations [30,31]. Furthermore, most IP-focused interventions were carried out in Western nations, did not concentrate on CRC, and disregarded the influence of SCs’ IPs on CPs as well as the consistency of IPs between couples. Johansson suggested that more research is needed to explore the nuances of the IPs of couples in order to comprehend the traits of IPs created during the coping process and refine couple-based IP interventions [27].

The preliminary effectiveness of IP interventions was substantiated, so it is prospective and meaningful to design IP-targeted interventions for dyads involving patients with CRC and SCs. Nevertheless, the current IP interventions were primarily formulated by professional norms and understandings of researchers rather than the true disease status and properties of the IPs of participants. A qualitative study examined the IPs of Chinese patients with CRC in relation to dietary changes but only targeted patients with CRC and disregarded the mutuality between couples and holistic coping experiences [21]. Thus, to develop and refine an IP intervention for couples dealing with CRC in a Chinese context, a deep qualitative interview was undertaken to explore IP features and IP interactions between the partners of couples and have a comprehensive understanding of their coping experiences.

## 2. Methods

### 2.1. Study Design

Interpretive phenomenology was chosen to be the approach in this study after prudently reviewing and identifying philosophical assumptions and claims. Each understanding of realities, which is based on the prior structure of primitive and changing understanding, is interpreted [32]. Heidegger claimed that the interpretation of a lived experience is foundational under descriptions of various contexts, and he used the term being-in-the-world to imply that experiencing lifeworld invariably affected the personal realities and narratives in which essences and meanings are embedded [33]. 

To understand the lived experiences of and strategies for coping with cancer, we have to explore the regular nature of the phenomenon through interpretive strategies and discover the interpretative understanding that targets the individual experiences of participants rather than instinctive descriptions. It is effective to interpret the essence and significance of experience based on one’s experience. Integrated with the objectives of this study, the selected method is appropriate.

### 2.2. Setting and Sampling

Purposive sampling was conducted to elect the most productive participants in oncological treatment centers and oncology wards in two hospitals in Wuxi, Jiangsu province, China from 2 November to 28 December 2023. 

The targeted subjects of this study were patients with CRC, SCs of patients with CRC, or couples dealing with CRC, and all of the participants were older than 18 years old. The marital relationships of the couples dealing with CRC were stable, and the patients with CRC admitted that their partners shouldered the majority of the caregiving burden. Both of them had been informed by the CRC diagnosis and had the basic abilities of understanding and expression and could coordinate well with the researchers’ interviews. The duration of the CRC diagnosis was not regarded as a restriction because the entire comprehension of IP combined with the coping experience in diverse cancer stages tends to produce considerate and multiform findings. 

Participants who had severe physical and mental diseases, such as major depression; patients who had memory, cognitive, and language dysfunction, which hampered the participants’ ability to correctly provide descriptions; and participants who were not interested in this study were excluded.

For the sampling procedure, the potential eligible participants were confirmed by retrieving the medical information of the hospitalized patients. Then, the prime researcher (Y.Z.) further approached them and verified the qualifications of the participants. The participants and researchers did not know each other previously. Each participant was invited and was explained the purpose and subject of the single face-to-face interview. After having enough time to think about it, informed consent was obtained from the participants and the convenient time was confirmed. All of the participants were promised that the interviews would not impact the normal treatment and care of their disease. The interviews were performed in the participants’ acquainted quiet oncological ward.

The collection of data was administered coincidently with the simultaneous analysis of data. When the 29th SC was included in this study, at which time authors Y.Z. and Y.W. jointly confirmed that no additional findings and concepts emerged, the data had reached saturation and purposive sampling was stopped.

### 2.3. Ethical Considerations

Ethical issues were considered in the entire process of this qualitative study [34]. This study was conducted in accordance with the ethical standards and regulations of the 2013 Declaration of Helsinki. The research attained ethical approval from the Human Ethics Committee of Jiangnan University (Approval NO. JNU202312IRB20) and the access of the involved hospitals. For the participants, written informed consent and oral consent prior to the beginning of the interview were obtained. The private information of the participants was anonymously processed, for example, the names were replaced by CP1, CP2, SC1, SC2, and so on, where CP represents cancer patient, and SC represents spousal caregiver. The transcripts were locked in a storage unit that was only accessible by the primary researcher. The participants were assured that they would not be identified in the subsequent spread of data and results.

### 2.4. Data Collection

The times of the interviews were selected by the participants, and researchers Y.Z. and Y.W., who are graduate students with expertise in qualitative research interviews, performed the face-to-face interviews. The interviews were recorded digitally. The countenance and body language were captured additionally in notes.

The interview guide was developed in a team conference, and team members included graduate students, senior oncology nurses, and psychological professors. Before the team discussion, all members jointly reviewed the literature on IP and reached a consistent understanding of the means of IP, which is the individual illness cognitive appraisal and emotional responses. To explore from which aspects of stance and perspective a person understood and viewed the illness and its influence, the first question was set. The first question asked what aspects and how the disease impacts participants to summarize the subjective perception towards the consequences of the disease and to aid the participants in recalling current and previous coping experiences. According to the study, it is known that feelings/emotions can influence the IPs of participants, and they are closely related to IP. In order to explore the created emotional responses towards the perceived threat of the disease and the methods the person used to deal with the negative effects of emotional distress on IP, we considered setting question 2. Additionally, it could be not disregarded that when facing the “disorder” roused by the negative event of cancer, the person spontaneously interpreted and gave meaning to the illness, namely cognition beliefs about cancer, which largely determined the modification models and outcomes of emotion regulation [35]. The cognitive beliefs towards the disease, which work on the IPs of participants paralleled with emotional responses, were also considered important parts that influence the formation and status of IP. In this regard, question 3 was set to ask about participants’ personal understanding and lay cognition about specific aspects of the disease (e.g., symptoms, treatment efficacy, and etiology) and the efforts and coping strategies they made to change their cognition during their coping experiences. To explore the deeper thoughts of the participants, question 4 was created to ask about individual meanings of the disease coping experience. It is known that conversations between couples modify their thoughts and feelings about the disease and coping experiences. In this regard, questions 5 and 6 were added to ask how participants communicate with their spouses about the disease and the emotional impacts of the communication and to elicit thoughts and reflection towards IP. To test the semi-structured interview guide and train the researchers, three pilot interviews were conducted. To supplement the insights and needs of the participants mentioned from pilot interviews to promote a positive IP, question 7 was added to help find directions for future supportive practices.

The semi-structured interview questions (Table 1), which were developed and confirmed after a conference between all authors and the testing and training of the three pilot interviews, guided each in-depth interview to stay relevant to the topics of research. A total of twenty-nine face-to-face interviews and two telephone interviews were conducted, and two telephone interviews were conducted as a second interview with one CP–SC pair and one SC to obtain additional interview information to ensure data completeness. The length of the interview time ranged from 23 to 67 min with an average of 30.2 min.

After the researchers clarified the unclear points and the meanings of the questions, further inquiries were addressed to the participants to cover the ambiguous answers. If the participants talked about the issues that were not related to the subject of the research, the researchers redirected them. At the end of the interviews, the participants were asked if there was something else they wanted to express and/or what kind of support and help could best benefit them to assist the researchers in discovering extra experiences and new thoughts. After each interview finished, the salient impressions and the typical features of the participants were promptly documented in memos. The recorded data were transcribed verbatim and instantly compared to the audio. 

### 2.5. Data Analysis

The findings of this study were concluded and reported in accordance with the Standards for Reporting Qualitative Research checklist [36]. An inductive thematic analysis, a common approach, was adopted in this study to generate themes [37,38]. NVivo 12 (QSR International) was utilized to aid in managing, coding, integrating subthemes, and producing themes. The researchers independently immersed themselves in the language context of the data to describe the insights by developing themes during which avoiding prejudice and taken-for-granted understanding as much as possible. Additionally, during the analysis process, we maintained regular self-reflection by regarding the subjective tendencies and attitudes, and after substituting ourselves as the participants in the disease and social context, we attempted to compare and integrate different opinions and experiences from broad and multi-faceted perspectives to confirm the objectivity of the data analysis. Member checks and specialist checks were conducted to help avoid individual researcher bias. The analysis outcomes were interpreted for the participants, and feedback was collected to identify and address the researcher bias. Discussions hosted by the corresponding author (Q.L., an experienced qualitative researcher and psychological counselor expert) were performed, which required iteratively weighing and considering the generated themes to resolve the dissensions and to reveal the multifaceted underlying meanings of the participants’ language.

### 2.6. Trustworthiness

To enhance the trustworthiness and credibility of the study’s results, several techniques were applied in the research. Each procedure was clearly and distinctly illustrated to strengthen the transparency of the study. The transcripts were individually checked by two prime researchers to ascertain the accuracy of the data. Using NVivo 12, a clear audit trail was maintained for the findings on the experiences of the participants since their diagnoses, which contributed to the dependability and transferability of the data. Brief presentations were conducted between the primary researchers to confirm the consistency of the data. The generated coding and themes were provided to the participants to ensure that the conclusions were correct and accurately reflected the thoughts and feelings of the participants coping with cancer. Additionally, peer debriefing was performed on the codes produced by two researchers (Y.Z. and Y.W.) to ensure the credibility and confirmability of the data analysis. To guarantee the validity of the results, the solid researcher triangulation comprised elementary researchers, senior clinical oncology nurses, and psychologists who determined the final themes.

## 3. Results

### 3.1. Characteristics of CPs and SCs

A total of 24 patients with CRC and 20 SCs participated in this study (15 CP-SC dyads, 9 CPs only, and 5 SCs only). The characteristics of the participants can be seen in Table 2. The more detailed demographic information of the CPs and SCs can be referred to in Tables S1 and S2. A total of 8 of 24 patients had stoma, 6 CPs were admitted for surgery, 14 CPs were admitted for chemotherapy, and 4 CPs were periodically admitted for re-examination.

Two themes were integrated and generated from the verbatim transcripts by iteratively reading and analyzing, including *individualized and predominant IP, IP sharing, and restructuring*. The subthemes and details are shown in Table 3.

### 3.2. Individualized and Predominant IP

#### 3.2.1. The Multiple Foundation of IP

(a) *Experiencing symptoms and active observation*

CPs and SCs initially formed the IP towards CRC by identifying the current symptoms of the disease and reflecting on the pre-disease behaviors to determine causes based on their disease knowledge and experiences. They noted that the main symptoms of CRC were hematochezia and stomachache, but they still had difficulty judging the severity of the disease by experienced symptoms. Lots of them recognized the severity of the disease and only perceived the disease strongly when they directly suffered or witnessed the treatment symptoms like extreme weakness and chemotherapeutic vomiting. As 5-CP said,

“*I just had a little blood in my stool, no pain, nothing else, so I didn’t know how serious it was; This is cancer after all, when I see these patients like me, they are suffering every day, I am really confused, very scared.*”

(b) *Previous and new illness knowledge*

With the rapid diagnosis and decision making in contemporary cancer settings, people (usually the tough partners in couples) who priorly communicated with prime physicians were well informed about the disease condition like the disease stage and progression, which formed their basic perceptions and psychological constructs, whereas persons (usually the vulnerable partners in couples) who preferred to distract themselves and SCs from stressful surroundings and reduce mental distress attempted to isolate themselves and use previous illness experiences and knowledge to understand the disease, by which basic perceptions and expectations of the illness were gradually formed. Like 12-CP-SC explained,

“*I had no idea about what was going on with her body at first. As the breadwinner of the family, I went to deal with her attending physician, and only after asking the physician in detail did I understand the disease. The stone in my heart was put down.” (12-SC); “I dared not ask him much about my illness. I guess it’s just an extra growth. At least it’s painful, so it shouldn’t be that bad.*”

(12-CP)

(c) *Vicarious experience from peers*

At the beginning of hospitalization, the couples dealing with CRC preferred to appraise and change their perceptions (especially for understanding the illness) by frequently interacting with ward mates who had similar ill conditions, including observing and interpreting their external reactions, like their expressions and tones, and their treatment statuses. The 10-CP who had a permanent stoma said the following:

“*The process was quick, and I was admitted to the hospital two days after the cancer diagnosis. It didn’t even hit me yet. Fortunately, the friends here are very warm. The woman who has had a permanent stoma for three years says that she is used to it. She showed me how to take care of the "pocket." In addition, I am a very optimistic person, so I did not suffer very much at the beginning, I believe that I will have a different future with this.*”

Hence, it could be summarized that the formation of basic perceptions of couples dealing with CRC was not only based on the experienced symptoms and feelings they identified but also on the verbal and vicarious experiences of others.

#### 3.2.2. The Natural Inconsistency of IP

(a) *Lay causal beliefs and role tendencies*

To understand the “chaos”, CPs and SCs attempted to interpret and raise some plausible etiologies for the illness, part of which were in conflict with the documented etiology. CPs were inclined to identify the disease as a consequence of external factors (e.g., environmental pollution and working stress), whilst SCs attributed the disease to internal factors (e.g., the inability of patients to take good care of themselves and unhealthy diet styles and habits). The differences in causal perceptions based on their standpoints and preferences were considerably apparent in the CRC populations, which could predict and explain the healthy and self-management behaviors of partners of patients. As 7-CP narrated,

“*When informed of the cancer diagnosis, I had already reflected on what could have caused the disease. There is a factory near my house. The discharged chemical gas and waste from it polluted the environment and also harmed my body. However, my wife has always disagreed with me, blaming and considering my hobbies (drinking, staying up late) as the root cause of my current bad situation.*”

(b) *The illness condition restrictions*

Regarding SCs, despite their desire to become the CPs’ salvation, the disease and treatment distress were intangible for them. Thus, this constricted condition directly caused a discrepancy within the identity of CRC, the prognosis, and the emotional reactions, which impacted the perceived duration and controllability of the illness. The number of identified symptoms related to SCs was less than that of CPs. The differences broadened, especially when the CPs were unable to clarify their discomfort sensations and mental symptoms, whereas the SCs’ perceptions of illness largely depended on the CPs’ cognitive and emotional presentations and knowledge from people around them (e.g., medical practitioners) who they trusted. Like 6-SC said,

“*I wish I had this disease so I could bear her pain. After her illness, she became more withdrawn and I didn’t know how to help her. I can’t really feel her state, I don’t know what kind of psychological pain she’s going through. I often ask the doctor if the medicine is effective, and search for some medical knowledge of the disease on the internet.*”

In summary, the CPs tended to attribute the disease to the causes that are uncontrollable, whilst the SCs had a desire to explain the disease regarding the patient-centered factors. Understanding the standpoints and expectations regarding the disease outcomes of different causal beliefs within couples dealing with CRC was important before attempting to match their IPs.

#### 3.2.3. The Significant Effects of IP Incongruence

(a) *Increasing psychological burden*

The match between the IPs of couples dealing with CRC reflected on the illness outcomes of the CPs and SCs to some extent. The CPs felt higher levels of distress and burden when their assessments of adjustment to CRC was incongruent with those of their partners, especially when the SCs believed that the healthcare staff had little control over the disease and that the consequences were severe. As 11-CP said,

“*In fact, in the past two years, I did not fully accept my illness, I did not completely withdraw from the pain. Endless chemotherapy and the review of the "verdict" reminded me again and again of those sufferings. I am so grateful that my husband accompanies me., but each time he asked me to face up, more stress occurred. He didn’t really understand me. I wanted him to understand my vulnerability and have more confidence in doctors. I think it’s rational to be a coward.*”

(b) *Emerging caregiving barriers*

Furthermore, the incongruence of IP increased the difficulty of spousal caring and dyadic coping. The SCs reported that the views and emotions of the CPs differing from theirs poses a challenge for them to identify the CPs’ underlying meanings and needs in a congruent format and then offer corresponding caring. Like 15-SCs explained,

“*I don’t understand how her crying all the time can help her condition. To me, the most important thing now is to consult multiple professional doctors as soon as possible to choose the most appropriate treatment plan. I tried to comfort her and cheer her up as soon as possible, but it didn’t seem to work.*”

(c) *Promoting individual temporary coping*

However, in some situations, the incongruence of IP appeared to be a signal of positive outcomes. The SCs found that their own views of the diseases’ reality were inconsistent with the CPs’ IPs. This revealed that the CPs created a distance to weaken the concept of the disease by separating themselves from the disease. The incongruence of IP indicated that the CPs had a good adjustment to cancer after active coping. Like 15-SC said,

“*I did everything on my own. She was also reluctant to reach out or ask questions about treatment options. Every time the disease came up, she refused to talk any further. I think it might be a protective mechanism for herself.*”

(d) *Assisting assess and providing tailored care support*

Moreover, the different extents of understanding the disease could be regarded as the adjusted outcomes based on the individualized coping styles of CPs and SCs. For example, some CPs and SCs searched for different kinds of information, whereas other CPs and SCs distracted themselves from illness-related information. After healthcare professionals assess the unique needs and preferences of individualized coping styles, they could receive appropriate informational support and reach to adapt the IP. As 19-SC narrated,

“*After so many years of cancer-fighting, it was not easy for me, or him. I don’t want to know too much about this disease, because it would make me panic and lose some confidence. But he always wanted to know more about the disease, and I didn’t stop him because I thought that I should respect his choice. I think it’s a different way for both of us to go forward.*”

Hence, the IP’s incongruence within couples dealing with CRC impacted their psychological adjustment to illness and life. It should be the case that a positive IP is transmitted between CPs and SCs rather than hopelessness and uncertainty. The advantages of there being differences in IPs among CPs and SCs should be recognized, and health promoters should intervene properly, where the IPs and diverse coping styles act as the intervention focus.

### 3.3. IP Sharing and Restructuring

#### 3.3.1. Leaving the CRC Diagnosis Behind

(a) *Focusing on apparent positive changes*

Couples dealing with CRC attempted to search for elements and ways to minimize the severity of the disease. Diversion coping strategies were commonly used to decrease negative IPs in couples dealing with CRC. They kept focusing on positive changes and emphasized the current health status, such as an apparent decreased index of cancer ligands, increased physical fitness ability, consolidated family connections, and so on. There is a notion that focusing on more positive aspects is a necessity for change, which could help an individual perceive a shorter timeline. As 13-CP said,

“*I think I’m a healthy person. Many people are afraid of it. But I was fine, there were no problems, I even forgot what happened to me.*”

(b) *Enhancing trust and diverting attention*

During the coping process, the IPs were self-assessed and restructured by the couples dealing with CRC. The trust and submissive attitude towards physicians could explain this. The beliefs of the treatment’s efficacy and the ability to control the disease were strengthened when they developed trust with their physicians and adopted almost all of the suggestions from their physicians without hesitation, which meant that they put themselves in the hands of their physicians. They also developed interests (e.g., performing light exercise and watching television) to take their minds off the CRC diagnosis. Like 10-CP narrated,

“*We have full trust in my attending. Medical science is so advanced now, that they must be able to cure my disease. I just need to work out, do what I love, and wait for my body to heal.*”

Couples dealing with CRC focused on moving forward and making efforts to improve the perceived consequences and expectations of the illness. Threatening IPs, which hampered psychological adaption, were gradually replaced by constructive IPs under their efforts and positive coping.

#### 3.3.2. Appropriate Disclosure and Reflection

(a) *Conversations about sensitive topics*

The difficulty in exchanging views and perceptions towards illness-sensitive topics was a common issue they experienced. Due to the specialty of the disease, especially for CPs who had stomas, social activities were reduced, and social dysfunction stood out. Additionally, the CPs tended to keep their emotions from the reach of their SCs and avoided disclosing negative feelings. Considering the extent of accepting CPs, the views about stoma caring and terminal topics were properly disclosed and discussed when a third person was present. In the process, not only were the thoughts about sensitive topics shared, but also the IP from the loved one, where the perceived ability of control was enhanced. As 8-SC said,

“*I understand that sometimes he doesn’t want to talk to me about this stoma, but it’s necessary to open the conversation at the right time.*”

(b) *Tentative expression at the right time*

Regardless of whether or not it was right, the SCs said that making CPs express their inner feelings and true thoughts about the disease treatment and caregiving was much more important. Their proper disclosure of the diagnosis and each communication before treatment made it easier to open the discussion about disease issues. The SCs treated proper disclosure as a first step to begin the conversation about the disease itself and indicated a positive attitude to coping with cancer together. Like 12-SC said,

“*To reassure him, the day before each chemo session, I would sit down and talk to him. In addition to letting him relax to bravely accept each chemotherapy challenge, I would like him to feel the confidence that I stand side by side with him.*”

(c) *Experience application and self-assessment*

Furthermore, recalling and reflecting improved the IPs of the CPs and SCs. They recalled past suffering or growth and generated precious experiences and enlightenment. They reflected on their weakness of health, colonoscopy awareness, and lack of basic medical knowledge in the past, leading to the progression of the disease. The CPs summarized the threshold of distress they could bear to ask for help and seek methods for alleviation. As 21-CP said,

“*I’ve never even heard of it before. I don’t get a colonoscopy for every physical. If I had known some basic health information then, I would be much better now.*”

It was the appropriate disclosure and reflection of experiences that developed the understanding and IPs of the CPs and SCs. CRC not only reinforced the psychological coping level when facing sudden traumatic events but also offered an opportunity to increase the level of cognition and analyzation. Furthermore, under the developed IPs, the priority of life was changed, and health and healthy behaviors were emphasized. 

#### 3.3.3. Information and Available Support

(a) *Obtaining understandable information*

From the diagnosis, treatment, and survivorship, the couples received a burst of information, which was difficult for them to remember and understand, especially when SCs needed to transfer massive amounts of illness-related information to the CPs and other family members. Some participants stated that the patients’ simpler explanations compared to those of the medical staff not only made them well-known about the disease the patients suffered from, but also alleviated their anxiety and disturbance, urging them to be more willing to participate in the treatment and care decisions. The parts of information that could be understood helped couples know exactly “what is it”, “what happened”, “what we would face”, and so on, increasing the confidence in the cure and control of the illness and decreasing perceived threats, thus improving the IPs. Like 8-CP said,

“*We are too old to understand the information around. The nurse did take a special paper and write it down and tell us what to do every step of the way, especially when we were first admitted, which made me feel warm.*”

(b) *Ab* *sorbing the new experience and consolidating stable IPs*

The other parts of information that could not be understood became overload information and an exogenous burden for them due to a lack of basic medical knowledge. For example, the SCs reported difficulty in independently dealing with large amounts of information and trifles at the initial stage, such as inpatient procedures, making decisions about the treatment scheme, and preparing for surgery. However, the IPs that were constructed with new concepts and gradually accepted knowledge were consolidated and difficult to be harmed by threatened symptoms and problems. As 16-SC narrated,

“*It was hard at first because she had a lot of fear about it because she didn’t know what to expect. But after six rounds of chemotherapy, she no longer had such strong emotional reactions.*”

(c) *Seeking external accessible support*

Besides obtaining knowledge from their own experiences, the patients also acquired information about managing treatment side effects and coping strategies from diverse avenues, including communicating with friends in ward mates and medical staff and reading illness-related brochures. However, as agents of CPs, the SCs undertook more negative IPs for the CPs and needed to face problems directly, especially regarding strict diet monitoring and ostomy care. For CPs, the SCs not only provided instrumental support (e.g., informed cancer diagnosis, timely appointed re-examination, and dietary supervision) but also offered some complementary illness-beneficial information like religious knowledge and advanced treatment regimens, which developed illness cognition, as 23-CP answered,

“*My husband not only prepares my meals carefully every day, but also learns something about Christianity. Every time we went to the hospital, he would tell me some stories about Jesus to soothe my heart.*”

## 4. Discussion

**Figure 1:**
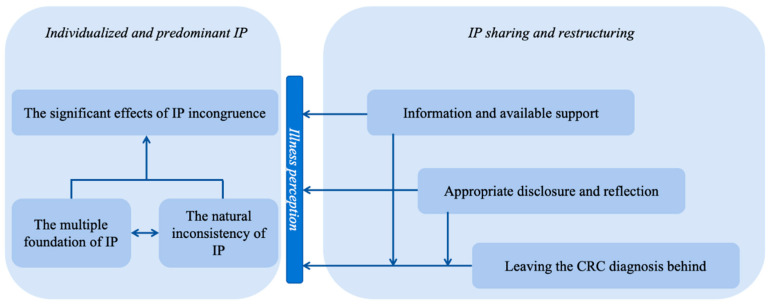
The preliminary framework of coping with IP among couples dealing with CRC.

This qualitative study explored the psychological traits of IPs among couples dealing with CRC and analyzed crucial coping strategies for threatening IPs. In order to clarify the relationships between six subthemes, a preliminary framework was generated (Figure 1). The left part of the framework reveals the significance and leading role of the IP’s psychological construct when coping with cancer among the partners of patients with CRC, whereas the right part of the framework presents their efforts in constructing adaptive IPs and adjusting to illness regarding promoting IP sharing and restructuring. This new framework could be used to explain and understand the characteristics of developed IPs under the interactions of couples dealing with CRC and their adopted coping approaches based on the perceived influence of the IP on coping experiences.

Each partner in the couples had a different role and undertook dissimilar responsibilities in the cancer trajectory [12]. They formed their basic perceptions towards the illness based on multiple sources (e.g., intrinsic sources of symptoms experienced and previous illness experiences or extrinsic sources of communication with physicians and ward mates), which determined the discrepancy compared to the way they perceived the illness at the beginning of its progression [27]. The unchangeable factors, like the ill-condition restrictions and stance of causes, suggested that there was a natural discrepancy within the IPs among couples [39], whereas the mutual impaction possessed between the above two aspects jointly works on the IPs’ incongruence and influences interactions among CPs and SCs. To make efforts to change maladaptive IPs, the three avenues they adopted promoted the sharing and restructuring of IPs in which the coping attitude of leaving the diagnosis behind reveals the major perception of illness while fighting cancer.

The fluctuation in IPs within the couples was impacted by the combination of intrinsic motivation based on their own hearts with extrinsic support and changed behaviors. The right and left parts build a cooperative relationship and mutual support paths. This mutual path not only provides an opportunity for partners to rationally determine their true illness statuses and individual thoughts, feelings, and needs, but also helps partners reach self-regulation and maintain the couples’ spontaneous collaboration during the cancer trajectory.

Except for learning about the illness experiences of ward mates, a professional support environment including psychology counselors, health promoters, and oncology specialists is beneficial for the stability and continuity of an optimistic IP status, especially for couples with major differences in coping attitudes. Having the ability to understand and interpret blasts of information became the common goal of couples and helped them amend their lay beliefs about the disease despite the discrepancy of tasks and tendencies in their current roles. The vital sources of change and coordination of the perception towards negative consequences and treatment beliefs were based on the observed responses of their spouses; for example, the SCs modified the IP according to the feedback and satisfaction of the CPs about caregiving, and the CPs changed the IP and expectations towards the disease based on the responses of the SCs to the disclosure of negative emotions.

Along with the study’s findings and preliminary framework, three aspects will be emphasized and discussed in detail, including adequate factual knowledge, positive congruence, and making meaning.

Providing adequate factual knowledge helped the couples better understand the current illness condition [40]. Previous findings also demonstrated that knowledge and information related to disease, which served as a changeable factor, effectively contributed to the understanding of the disease and IP [41]. They acknowledged the importance of obtaining useful and detailed information from medical staff about CRC to form a basic IP before the beginning of treatment. However, the participants stated that despite rapid diagnosis and treatment saving a large amount of time, the time constraints of the physicians made them realize their lack of factual and scientific knowledge about the disease and the instability level of the IP, causing a high level of anxiety. The internet and communication with ward mates became the limited sources of knowledge that they used to understand the symptoms and experiences they were confronting, whilst the couples expressed concern about the reliability and authenticity of the knowledge [17]. Timely knowledge offered by professional staff could reduce concerns about the treatment’s effects and encourage the adherence to medical treatment after addressing the erroneous beliefs. Thus, transferring factual knowledge from health professionals to couples in spite of it resulting in a negative conceptualization about the illness could promote a coherent understanding of the illness and promote better adjustment. Additionally, the two extreme coping styles regarding illness-related knowledge existed among couples, namely seeking all kinds of information and distracting themselves from information [40]. Thus, the provision of adequate factual knowledge is not only in line with the needs they perceive but also in accordance with different knowledge coping styles. The improvement in dissimilar IP coherence relied on individualized information provision and expanding sources of factual knowledge.

Positive congruence played a significant role in the IPs of CPs and SCs, which suggested that, except for reaching a consensus and congruence, the IPs of CPs and SCs all need to develop positively and adjust to cancer. In particular, three dimensions of IPs, including the timeline, coherence (global comprehension of illness), and concern of illness, were identified as the critical points for positively impacting personal IP and matching the IPs of CPs and SCs smoothly. The complex mutual interactions between CPs and SCs were not adequately verified. However, the CPs reported more psychological distress and lower QOL when they perceived that their partners had short timeline perceptions, less concern, and less comprehension about the disease [42]. This could be explained by the fact that the CPs felt that the SCs minimized the severity of the illness [24]. For a person, the timeline perception, coherence, and concern about the illness determine the identification of symptoms and illness and cure/control beliefs, the severity of perceived negative consequences, and emotional status. This notion echoed the result of previous research stating that a pessimistic IP is closely correlated with negative emotions (depression and anxiety) and negatively impacts the QOL of cancer populations [43]. Guiding the spouses’ emotional support and negative emotion disclosure contributed to emotion recovery and IP regulation. It was recognized that regardless of the level of IP, it was the positive match between the CPs’ and SCs’ adaptive IP towards cancer that made the participants feel united with their partners rather than experience conflict and disagreement, especially for CPs.

Making meaning was regarded as the ultimate achievement in terms of CRC perceptions about illness and coping experience [44]. The beliefs of the IP and goals coordinated into making meaning, which comprises illness meaning and life meaning. The personal IP system was improved and refined in line with making meaning, which could buffer the coping, appraisal, and interpretation of stress [45]. Differing from negative brooding, proactive recalling and reflection concerning positive change during cancer coping promoted the reframing of life meaning after traumatic events. Thus, the way the participants perceived the illness was changed, and they reappraised the illness and assigned a new meaning to the illness. The person who positively perceived illness actively managed stressful situations, focusing on positive interpretation and the reconstruction of difficult experiences. This was consistent with a previous research result in which the accumulation of successful experiences encouraged positive thoughts towards the disease expectations and increased confidence in facing a challenge of similar difficulty [25]. In parallel with the reconstruction of life meaning and illness meaning, the beliefs of IP were gradually improved as the disease progressed.

To conclude, the findings of this qualitative study present potential recommendations for IP intervention in a couple of dyads, namely adequate factual knowledge, positive congruence, and making meaning. Although the targeted population of this study was couples dealing with CRC, we thought that guiding the illness’s meaning and promoting positive IP congruence in couples coping with disease could be applied to other cancer types and chronic illnesses. The influence of disease on the subjective physical, mental, and social functions became a consideration for modification in terms of providing factual knowledge. Considering the differences in the characteristics of the disease and its influence, it is important to research the specific disease and obtain more common findings in more research. This study also has some limitations. On the one hand, when accounting for illness condition restrictions, the interviewers could not completely and precisely “translate” and optimize the narrations from the participants. On the other hand, referring to the influence of sociocultural context, the identity considerations and social norms limited the adequate expression of inner thoughts. Additionally, all of the participants came from Wuxi City in China, which limits the generalizability of the findings to other regions. Hence, to obtain more results and findings, more research could be conducted in various regions targeting diverse cancer types. 

When the IPs of the partners of patients vary largely, the problems during cancer trajectory will be exposed faster and more pronounced, and couples dealing with CRC will feel more flustered and helpless and need professional support. We advocated that developing easily accessible IP interventions can help couples positively view and communicate their differences in thoughts and feelings towards cancer. Health promoters should evaluate the IPs of partners of couples in a timely manner and confirm the positive effects of developing the IP. By combining their experiences, the findings will contribute to the development of IP interventions for couples dealing with CRC. In addition, it may increase IP program acceptance so that CRC benefits from the intervention.

Based on the findings, we advocate that prior to receiving healthcare, evaluating previous disease experiences and the expectations of medical care is beneficial to predict and identify the IP traits of couples and formulate effective tailored support. Couples who have poor health before the CRC diagnosis are prepared with the aim of alleviating aggravating emotional responses and more negative thoughts towards the disease in the initial stage.

## 5. Conclusions

This study investigated the dyadic relationship between IP and couples coping with CRC, filled the gap regarding the IP and coping experiences of couples dealing with CRC, and contributed to the improvement of the IP program. It was also the first qualitative study to explore the features of IP and coping experiences from the perspectives of CRC dyads, which formed the psychological construct of IP and helped analyze relationships among six subthemes and how they jointly contribute to the adjustment of the IP and the promotion of illness outcomes. Furthermore, combined with previous findings, this study furnished potential evidence and recommendations for developing a couple-based IP intervention. Each component of the framework and three vital aspects (e.g., adequate factual knowledge; the congruence of concern, timeline, and coherence; and making meaning) became useful suggestions for health professionals to help reduce threatening IPs and reach coherence between CPs and SCs’ IPs. In the early stage, expanding sources of information and providing adequate factual knowledge are carried out with the aim of helping couples become well prepared for the current and subsequent difficulties. In addition to providing effective extrinsic support, helping couples apply knowledge and skills and facilitating the absorption of experiences, during which the self-driven force is enhanced, are the adjustments that require major attention in the care program of health promoters. Encouraging a mutual open conversation regarding disease-centered topics (e.g., course, symptoms, and negative consequences) is important to promote the positive congruence of IPs in the middle stage. Before the conversation, improving the sensitivity of a partner to perceive their spouse’s feelings and various thoughts is beneficial to promote the positive congruence of the IP. Viewing setbacks with optimism and recalling illness meaning are strategies that aim to maintain the stability of a positive IP in long-term survivorship.

## Figures and Tables

**Table 1:** Semi-structured guides for each in-depth interview.

Questions for Patients with CRC, Their SCs, or Couples Dealing with CRC
How do you think about that the disease has affected you? How do you deal with the disease and the challenges? How does the disease make you feel? How has it changed? What are the possible impacts? Have your knowledge and understanding of the disease changed? How has it improved? How might it have been affected? What does the illness mean to you? How do you and your partner communicate about the illness and emotions? What do you think positive IP is? How do you think about your IP? What kind of help and support would you like?

**Table 2:** The characteristics of the patients with CRC and SCs.

Characteristics	CPs	SCs
Gender (n, %)	Male (13, 54.2%); female (11, 45.8%)	Male (8, 40%); female (12, 60%)
Mean age (y)	57.8 (range, 32–70)	58.8 (range, 36–69)
Cancer types (n, %)	Colon cancer (17, 70.8%)	
	Rectal cancer (7, 29.2%)	
Mean time since diagnosis (m)	17.4 (range, 1–60)	
Average length of time in their role as SC		13.2 m (range, 1 m–3 y)
Levels of education (n, %)	Undergraduate education (9, 37.5%) Middle school (9, 37.5%) Primary school (6, 25%)	Undergraduate education (6, 30%) Middle school (10, 50%) Primary school (4, 20%)
Informed about disease (n, %)	Well informed (13, 54.2%) Partly informed (11, 45.8%)	Well informed (12, 60%) Partly informed (8, 40%)
Total (n)	24	20

Annotation: CPs, cancer patients; SCs, spousal cancers; n, number of cases; y, year; m, month.

**Table 3:** A framework of themes and subthemes.

Themes	Subthemes
Individualized and predominant IP	The multiple foundations of IP
	The natural inconsistency of IP
	The significant effects of IP incongruence
IP sharing and restructuring	Leaving the CRC diagnosis behind
	Appropriate disclosure and reflection
	Information and available support

## Data Availability

We (the authors) have full control of all primary data and agree to allow the journal to review the data if requested. The data are available upon request from the corresponding author. The data are not publicly available due to the participants’ personal information being included.

## Supplementary Materials

The following supporting information can be downloaded at: https://www.mdpi.com/article/10.3390/healthcare12111073/s1, Table S1: The detailed demographic characteristics of CRC patients. Table S2: The detailed demographic characteristics of CRC SCs.
