# Supplementary material for: The Illness Perceptions and Coping Experiences of Patients with Colorectal Cancer and Their Spousal Caregivers: A Qualitative Study
Source: Healthcare (Basel). 2024 May 24;12(11):1073. doi: 10.3390/healthcare12111073 (PMC11171850; doi:10.3390/healthcare12111073)
Supplement: Supplementary file 1 [file healthcare-12-01073-s001.zip › healthcare-2982222-supplementary.pdf]

Table S1: The detailed demographic characteristics of CRC patients.

Number	Age	Gender	Cancer types	Cancer stage	Duration of illness (months)	Levels of education	Informed about the disease	Having stoma or not (yes or no)	Treatment stage
1CP	64	F	RC	Stage III	2	primary school	partly informed	Yes	Surgery
3CP	58	F	CC	Stage III	37	undergraduate education	well informed	No	Chemotherapy
4CP	59	F	CC	Stage II	25	primary school	well informed	No	Chemotherapy
5CP	61	M	CC	Stage III	23	undergraduate education	well informed	No	Chemotherapy
6CP	36	F	CC	Stage II	7	undergraduate education	partly informed	No	Surgery
7CP*	32	M	CC	Stage III	48	undergraduate education	well informed	No	Chemotherapy
8CP	52	M	CC	Stage III	5	middle school	partly informed	Yes	Chemotherapy
9CP*	58	M	RC	Stage III	37	middle school	well informed	Yes	Reexamination
10CP	63	M	RC	Stage III	11	primary school	well informed	Yes	Chemotherapy
11CP	67	F	CC	Stage IV	38	primary school	partly informed	No	Chemotherapy
12CP	65	M	RC	Stage III	10	undergraduate education	well informed	Yes	Reexamination
13CP*	67	M	RC	Stage III	36	middle school	well informed	Yes	Chemotherapy
15CP	59	F	CC	Stage III	1	middle school	partly informed	No	Surgery
17CP*	47	F	CC	Stage III	4	middle school	partly informed	No	Surgery
18CP	70	M	CC	Stage IV	10	undergraduate education	well informed	No	Chemotherapy
19CP	69	M	CC	Stage III	12	undergraduate education	well informed	No	Chemotherapy
20CP*	51	F	CC	Stage II	1	middle school	partly informed	No	Surgery
21CP*	37	F	CC	Stage II	2	middle school	partly informed	No	Surgery
22CP	60	M	CC	Stage III	2	primary school	partly informed	No	Chemotherapy
23CP*	66	F	RC	Stage IV	3	primary school	partly informed	Yes	Chemotherapy
24CP*	62	M	RC	Stage IV	18	undergraduate education	well informed	Yes	Chemotherapy
25CP	54	M	CC	Stage III	24	middle school	well informed	No	Reexamination
26CP	67	F	CC	Stage III	2	undergraduate education	partly informed	No	Chemotherapy
27CP*	64	M	CC	Stage III	60	middle school	well informed	No	Reexamination

Legends: Abbreviations: CP: cancer patient; *: only cancer patient; M: male; F: female; CC: colon cancer; RC: rectal cancer.

Table S2: The detailed demographic characteristics of CRC SCs.

Number	Age	Gender	The length of caregiving time (months)	Levels of education	Informed about the disease
1SC	65	M	2	middle school	partly informed
2SC*	53	F	12	undergraduate school	well informed
3SC	59	M	36	undergraduate school	well informed
4SC	58	M	25	middle school	well informed
5SC	57	F	23	undergraduate school	well informed
6SC	36	M	6	middle school	partly informed
8SC	51	F	5	middle school	partly informed
10SC	60	F	11	middle school	well informed
11SC	67	M	36	middle school	well informed
12SC	66	F	10	undergraduate school	well informed
14SC*	65	F	11	primary school	well informed
15SC	58	M	1	middle school	partly informed
16SC*	56	M	2	primary school	partly informed
18SC	67	F	10	undergraduate school	well informed
19SC	69	F	12	middle school	well informed
22SC	59	F	2	primary school	partly informed
25SC	57	F	22	primary school	partly informed
26SC	65	M	2	middle school	well informed
28SC*	51	F	3	middle school	partly informed
29SC*	57	F	31	undergraduate school	well informed

Legends: Abbreviations: SC: spousal caregiver; *: only spousal caregiver; M: male; F: female.
